# Editorial: Pharmacogenomics and ethnicity: Prevalence and clinical significance of pharmacogenomic biomarkers in indigenous and other populations

**DOI:** 10.3389/fphar.2023.1180487

**Published:** 2023-03-29

**Authors:** George P. Patrinos, Luis Abel Quinones, Chonlaphat Sukasem

**Affiliations:** ^1^ Laboratory of Pharmacogenomics and Individualized Therapy, Department of Pharmacy, School of Health Sciences, University of Patras, Patras, Greece; ^2^ Department of Genetics and Genomics, College of Medicine and Health Sciences, United Arab Emirates University, Al-Ain, United Arab Emirates; ^3^ United Arab Emirates University, Zayed Center for Health Sciences, Al-Ain, United Arab Emirates; ^4^ Laboratory of Chemical Carcinogenesis and Pharmacogenetics (CQF), Department of Basic and Clinical Oncology, Faculty of Medicine, University of Chile, Santiago, Chile; ^5^ Latin American Network for Implementation and Validation of Clinical Pharmacogenomics Guidelines (RELIVAF-CYTED), Madrid, Spain; ^6^ Division of Pharmacogenomics and Personalized Medicine, Department of Pathology, Faculty of Medicine Ramathibodi Hospital, Mahidol University, Bangkok, Thailand; ^7^ Pharmacogenomics and Precision Medicine Clinic, Bumrungrad Genomic Medicine Institute (BGMI), Bumrungrad International Hospital, Bangkok, Thailand

**Keywords:** pharmacogenomics, pharmacogenetics, personalized medicine, population, genetics

Ethnicity and pharmacogenomics are inextricably linked, and drug responses can vary based on the allelic distribution present in different ethnic populations. For instance, the CYP2C9*2 allele prevalence is <1% in most Asian populations, while it can reach up to 19% in some European populations. In some countries, due to different waves of immigration and admixtures, dissimilar genetic patterns are observed. This interethnic genetic heterogeneity may have a significant impact when it comes to drug responses in terms of efficacy and safety. Recent advances in the field of population genomics reinforce the perception that population genetic diversity is the next big thing in personalized medicine, namely population pharmacogenomics, where a series of pharmacogenomics data can be exploited within public health (Lakiotaki et al., 2017; Patrinos, 2018).

Unfortunately, most of the time, only major ethnic groups are of interest for investigations aiming to discover clinically actionable pharmacogenes. Importantly, ethnicity can also influence socioeconomic status, which can lead to inequitable healthcare support for many ethnic minorities around the world. This could be the reason why the results of any given pharmacogenetics study are not being applied as they could be in general clinical practice. A very characteristic example is the HLA B*15:02 variant that has been associated with carbamazepine-induced SJS/TEN in general, but in Malaysia, HLA B*15:13 has been identified as the next potential ethnic-specific risk marker for carbamazepine-induced SJS/TEN (Sukasem et al., 2018). In addition, in Latin America, arguably the largest recently mixed population among European, African and Amerindians is underrepresented in world databases (Salzano and Sans, 2014; STATISTA, 2022). This issue and other barriers for personalized medicine could explain that pharmacogenomics are only scarcely available in this region (Quinones et al., 2014).

Consequently, it is of paramount importance that we determine and establish a specific map for population-specific pharmacogenomic biomarkers that have the potential to directly impact and promote the clinical implementation of pharmacogenomics in very specific and unrepresented populations.

This Research Topic aimed to present and outline recent evidence dealing with ethnic-specific pharmacogenomics biomarkers, as well as their therapeutic relevance in various ethnicities and to explore the ethnic-specific genetic complexity and how it can influence the implementation and the use of ethnically unique pharmacogenes in clinical practice.

It is well established that carbamazepine can trigger dermatologic hypersensitivity reactions, associated with specific human leukocyte antigens (HLAs), especiallyHLA-B*15:02 and HLA-A*31:01. In their article, Fernandes et al. have explored the predictive performance of the rs1061235 and rs17179220 variants as HLA-A*31:01 proxies in several native American (sub)populations, that are largely underrepresented in several pharmacogenomic studies, such as the Native American population of the Human Genome Diversity Project (HGDP), Kaingang and Guarani adults from indigenous reservation areas in Brazil, among others. The range of the frequencies observed, namely HLA-A*31:01 (0.02–0.65), rs1061235 (0.03–0.13) and rs17179220 (0.12–0.66), is indicative of the diversity of cohorts employed. Overall, this study indicates that rs1061235 and rs17179220 are not optimal proxies for HLA-A*31:01in Native American populations.

Similarly, data on the prevalence of *HLA-B* pharmacogenomics biomarkers are also scarce in the Arab countries. Dashti et al. investigated the frequencies of major *HLA-B* pharmacogenomics biomarkers in the Qatari population. In particular, these authors have emoployed next-generation sequencing data from 1,098 Qatari individuals for *HLA-B* typing. Furthermore, *HLA-B* pharmacogenomics biomarkers were obtained from the HLA Adverse Drug Reaction Database. It was shown that the HLA-B*51:01 variant is the most frequent pharmacogenomics biomarker (26.67%) in the Qatari population, associated with phenytoin- and clindamycin-induced ADRs. The second most frequent pharmacogenomics biomarker is the HLA-B*58:01 allele (6.56%), which is associated with allopurinol-induced ADRs. These *HLA-B* biomarkers can contribute towards the establishment of a pharmacogenomics screening program in Qatar for cost effective interventions aimed at preventing drug-induced hypersensitivity.

Life-threatening severe cutaneous adverse reactions, such as drug reaction with eosinophilia and systemic symptoms (DRESS) can be caused by vancomycin, which is a commonly used antibiotic. These symptoms have been strongly associated with the HLA-A*32:01 variant in populations of European ancestry. Wang et al. sought to explore the genetic predisposition of vancomycin-induced DRESS in the Han-Chinese population. In a rather small sample of patients with vancomycin-induced DRESS (n-26), patients tolerant to vancomycin (n-51) compared with a large number of general population controls (*n* = 1616), it was shown that vancomycin-induced DRESS was associated with HLA-A*32:01, HLA-B*07:05, HLA-B*40:06 and HLA-B*67:01. As a result, this study identified additional pharmacogenomics biomarkers for vancomycin-induced DRESS in the Han-Chinese population.

On the other hand, the enzyme cytochrome P450 2D6 (CYP2D6) metabolises approximately 20%–25% of commonly prescribed drugs, including analgesics, anti-hypertensives, and anti-depressants, among others. It is well established that *CYP2D6* genetic variants can affect the metabolizing rate of several drugs, including predisposing to adverse drug reactions. Several indigenous and minority populations, such as those from Oceania, were greatly underrepresented in research pertaining to the *CYP2D6* gene variant prevalence, which can have importance clinical significance. In this respect, Hitchman et al. examined the frequency of *CYP2D6* variants in a sample of 202 Māori and Pacific individuals, living in Aotearoa (New Zealand). By using a combined long PCR-based/nanopore sequencing approach, they identified all variants and alleles in these samples. These authors identified twelve variants previously not reported in the PharmVar *CYP2D6* database, from which three were exonic missense variations. Importantly, the CYP2D6*71 allele, a variant of uncertain functional status which has been rarely observed in previous studies, was identified at a relatively high frequency (8.9%) within this cohort. Thus, these data will contribute towards accurate and effective *CYP2D6* pharmacogenomics analysis in this population group.

Overall, the contents of the articles in this Research Topic further highlight the need to expand our pharmacogenomics research from the individual to the population-specific level, focusing in particular on underrepresented populations. This would help to further overcome those obstacles where healthcare decisions can be made at the population level, making the concept of population pharmacogenomics from variant identification to clinical implementation, more feasible, especially coupled with the necessary economic evaluations (Simeonidis et al., 2019), particularly for developing countries.
